# Onionin A inhibits ovarian cancer progression by suppressing cancer cell proliferation and the protumour function of macrophages

**DOI:** 10.1038/srep29588

**Published:** 2016-07-12

**Authors:** Junko Tsuboki, Yukio Fujiwara, Hasita Horlad, Daisuke Shiraishi, Toshihiro Nohara, Shingo Tayama, Takeshi Motohara, Yoichi Saito, Tsuyoshi Ikeda, Kiyomi Takaishi, Hironori Tashiro, Yukihiro Yonemoto, Hidetaka Katabuchi, Motohiro Takeya, Yoshihiro Komohara

**Affiliations:** 1 Department of Cell Pathology, Graduate School of Medical Sciences, Kumamoto University, Honjo 1-1-1, Chuo-ku, Kumamoto 860-8556, Kumamoto, Japan.; 2 Obsterics and Gynecology, Graduate School of Medical Sciences, Kumamoto University, Honjo 1-1-1, Chuo-ku, Kumamoto 860-8556, Kumamoto, Japan; 3 Department of Natural Medicine, Faculty of Pharmaceutical Sciences, Sojo University, Ikeda 4-22-1, Nishi-ku, Kumamoto 860-0082, Japan; 4 Department of Mother-Child Nursing, Faculty of Life Sciences, Kumamoto University, Kuhonji 4-24-1, Chuo-ku, Kumamoto 862-0976, Kumamoto, Japan; 5 Priority Organization for Innovation and Excellence, Kumamoto University, Kurokami 2-39-1, Chuo-ku, Kumamoto 860-8555, Kumamoto, Japan

## Abstract

It is well known that tumour-associated macrophages (TAMs) play an important role in tumour development by modulating the tumour microenvironment, and targeting of protumour activation or the M2 polarization of TAMs is expected to be an effective therapy for cancer patients. We previously demonstrated that onionin A (ONA), a natural low molecular weight compound isolated from onions, has an inhibitory effect on M2 macrophage polarization. In the present study, we investigated whether ONA had a therapeutic anti-ovarian cancer effect using *in vitro* and *in vivo* studies. We found that ONA reduced the extent of ovarian cancer cell proliferation induced by co-culture with human macrophages. In addition, we also found that ONA directly suppressed cancer cell proliferation. A combinatorial effect with ONA and anti-cancer drugs was also observed. The activation of signal transducer and activator of transcription 3 (STAT3), which is involved in cell proliferation and chemo-resistance, was significantly abrogated by ONA in ovarian cancer cells. Furthermore, the administration of ONA suppressed cancer progression and prolonged the survival time in a murine ovarian cancer model under single and combined treatment conditions. Thus, ONA is considered useful for the additional treatment of patients with ovarian cancer owing to its suppression of the protumour activation of TAMs and direct cytotoxicity against cancer cells.

Epithelial ovarian cancer (EOC) is one of the most lethal female cancers in the world. Although the number of new cases of EOC ranked tenth among female malignancies, the number of deaths due to EOC ranked fifth in the United States^1^. Clinically, peritoneal dissemination and ascitic fluid are common clinical features of advanced EOC, which are not only difficult to excise using surgery but also often resistant to chemotherapy. In other words, one of the keys in the treatment of patients with EOC is controlling peritoneal dissemination and ascitic fluid. It is well known that the cancer microenvironment in the peritoneal cavity is important for EOC progression^2^. Many infiltrating macrophages (referred to as tumour-associated macrophages, TAMs) are detected in the primary lesion and ascitic fluid of patients with advanced EOC, and TAMs are considered to play critical roles in the development of peritoneal dissemination^3^,^4^,^5^,^6^.

Recent studies revealed heterogeneity in macrophage function. Many researchers suspect that macrophages can differentiate into various activation states owing to the cytokine balance in the microenvironment. Briefly, macrophages are differentiated into the M1 (classically activated) phenotype by Th1-type cytokines or bacterial products and are differentiated into the M2 (alternatively activated) phenotype by Th2-type cytokines. We previously demonstrated that nearly all TAMs in the primary lesions and ascites of patients with EOC are polarized towards the M2 phenotype, which has a protumour function^6^,^7^. Furthermore, *in vitro* co-culture experiments have shown that the activation of signal transducer and activator of transcription 3 (STAT3), which plays an important role in tumour progression and chemo-resistance in EOC cells, was strongly induced by co-culture with M2 macrophages^6^,^8^,^9^. M2 macrophages activated by direct contact with EOC cells secrete several cytokines such as IL-6 and IL-10, which in turn induced tumour cell activation. Activated M2 macrophages are also considered to be related to angiogenesis, tumour invasion, tumour metastasis, and immunosuppression^10^,^11^,^12^,^13^,^14^. Therefore, macrophage polarization into the M2 phenotype and the cell-cell interaction of M2 macrophages and tumour cells are believed to be emerging targets to block EOC progression.

We have previously attempted to identify natural compounds that inhibit macrophage polarization into the M2 phenotype^15^,^16^,^17^,^18^,^19^, and we identified onionin A (ONA), a new natural compound containing sulfur that is isolated from onions^20^. In the present study, we examined whether ONA has a beneficial effect and/or a combinatorial effect with chemotherapy for EOC using both *in vitro* and *in vivo* studies.

## Results

### ONA inhibits the cell-cell interaction between M2 macrophages and EOC cells

**Figure 1:**
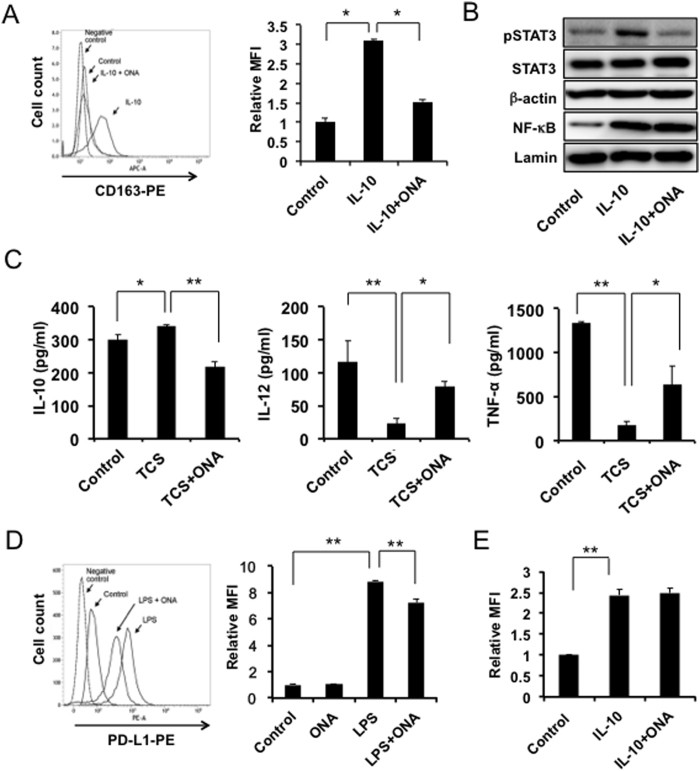
Effect of ONA on surface molecules and cytokine secretion in HMDMs. Human monocyte-derived macrophages (HMDMs) were stimulated with IL-10 in the presence of DMSO or ONA (30 μM) for 24 hours. The CD163 expression was evaluated by flow cytometry (**A**) and the activation of STAT3 and NF-κB was evaluated by a Western blot analysis, as described in the Materials and Methods (**B**). HMDMs were stimulated with LPS (100 ng/ml) for 24 hours after incubation with ONA (30 μM) for 24 hours in the presence of TCS, followed by determination of the levels of IL-10, IL-12 and TNF-α secretion using ELISA, as described in the Materials and Methods (**C**). HMDMs were stimulated with LPS (**D**) or IL-10 (**E**) in the presence of DMSO or ONA (30 μM) for 24 hours, and the PD-L1 expression was evaluated by flow cytometry, as described in the Materials and Methods. The data are presented as the mean ± SD. *p-value < 0.05, **p-value < 0.01 vs. control.

First, we determined whether ONA inhibited the EOC cell-induced M2 polarization of human monocyte-derived macrophages (HMDMs), as described in our previous study. As shown in Fig. 1A, CD163 overexpression induced by IL-10 stimulation was significantly abrogated by ONA. ONA inhibited STAT3 activation, whereas NF-κB signalling was not influenced (Fig. 1B).

Tumour culture supernatant (TCS) increased the secretion of IL-10, one of the M2 phenotype markers, and decreased the secretion of IL-12 and TNF-α, M1 phenotype markers, in the HMDMs. Under the assay conditions used, ONA significantly suppressed TCS-induced IL-10 secretion and enhanced the IL-12 and TNF-α secretion reduced by the TCS (Fig. 1C). PD-L1 is well known to be involved in immunosuppression, and we found that ONA abrogated the PD-L1 overexpression induced by LPS stimulation (Fig. 1D). IL-10 is also known to induce PD-L1 overexpression; however, IL-10-induced PD-L1 upregulation was not inhibited by ONA (Fig. 1E). These data indicated that ONA inhibited the EOC cell-induced M2 polarization of HMDMs.

**Figure 2:**
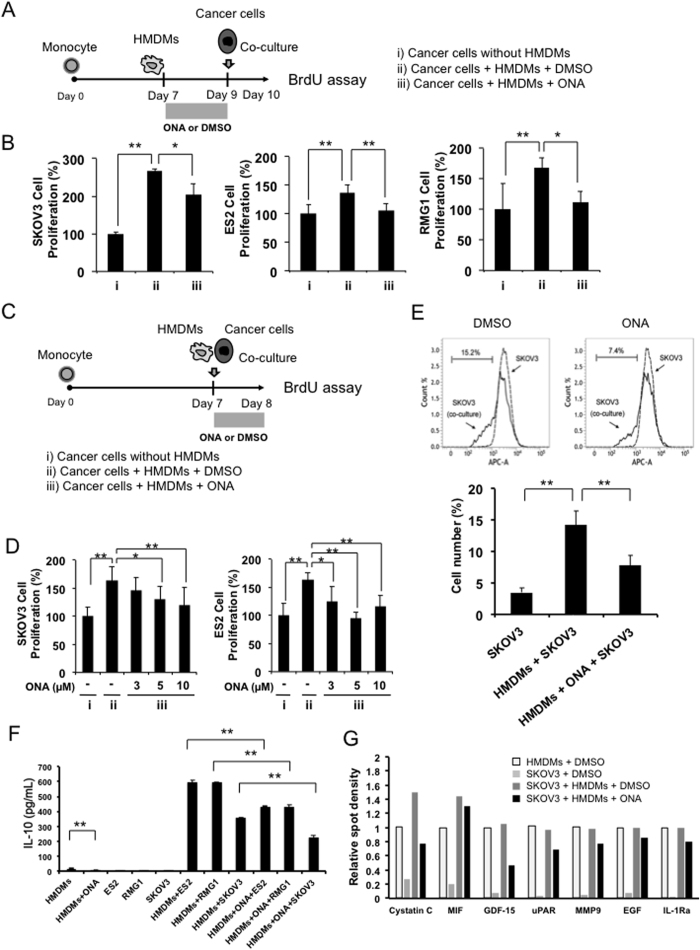
Effect of ONA on tumour cell proliferation in co-culture of EOC cells with HMDMs. Compared with EOC mono-culture (**A**-i), non-treated (**A**-ii) and onionin A (ONA) (10 μM)-treated HMDMs (**A**-iii) were incubated with epithelial ovarian cancer (EOC) cells (SKOV3, ES2, and RMG1) for 24 hours, followed by the determination of BrdU-positive cells by ELISA (**B**). Compared with EOC mono-culture (**C**-i) and co-culture without ONA treatment (C-ii), co-cultured cells (HMDMs and EOC cells) were treated with the indicated concentrations of ONA (3–10 μM) for 24 hours (**C**-iii), followed by the determination of BrdU-positive cells by ELISA (**D**). SKOV3 cells were labelled with CytoTell Red fluorescence and co-cultured with HMDMs, and then cell proliferation was assessed by flow cytometry (**E**). IL-10 production was evaluated by ELISA (**F**). HMDM-derived soluble factors were evaluated by a cytokine array kit, and the relative spot densities of cystatin C, macrophage migration inhibitory factor (MIF), growth differentiation factor (GDF)-15, urokinase plasminogen activator receptor (uPAR), matrix metalloproteinase 9 (MMP9), epidermal growth factor (EGF), and IL-1 receptor antagonist (IL-1Ra) were evaluated using the ImageJ software programme (**G**). The data are presented as the mean ± SD. *p-value < 0.05, **p-value < 0.01 vs. control.

Because it is well known that activated M2 HMDMs accelerate cancer cell proliferation, we hypothesized that ONA inhibited the cell-cell interactions between macrophages and cancer cells. To investigate the inhibitory effects of ONA on cell-cell interactions, we examined the effect of ONA on the interaction between EOC cells and HMDMs, as shown in Fig. 2A. The proliferation of EOC cells was significantly increased by co-culture with macrophages (p-value < 0.01 in all cell lines), and this protumour function of the HMDMs was suppressed (p-value < 0.05 in SKOV3 or RMG1 and <0.01 in ES2) by ONA treatment (Fig. 2B). Furthermore, we measured the effect of ONA on the proliferation of EOC cells in co-culture conditions resembling actual EOC patients, as shown in Fig. 2C. ONA treatment also inhibited EOC cell proliferation under this model, even if we administered ONA after initiating co-culture with EOC cells and HMDMs (Fig. 2D,E). To test the effect of ONA on HMDM activation, we next investigated cytokine production using an ELISA and cytokine array kit as described in the materials and methods. Because IL-10 is preferentially produced by activated HMDMs, and most cancer cell lines secrete no or low concentrations of IL-10, IL-10 production is useful to evaluate HMDM activation. As shown in Fig. 2F, IL-10 production was strongly induced by the co-culture condition, and IL-10 production was significantly suppressed by ONA. As shown in Fig. 2G and Supporting Information 1, soluble molecules (cystatin C, macrophage migration inhibitory factor, growth differentiation factor-15, urokinase plasminogen activator receptor, matrix metalloproteinase 9, epidermal growth factor, and IL-1 receptor antagonist) considered to be secreted from HMDMs were found to be suppressed by ONA. These data indicated that ONA inhibited the proliferation of EOC cells by abrogating HMDM activation induced by cell-cell interaction.

### ONA inhibits EOC cell proliferation by suppressing STAT3 activation

**Figure 3:**
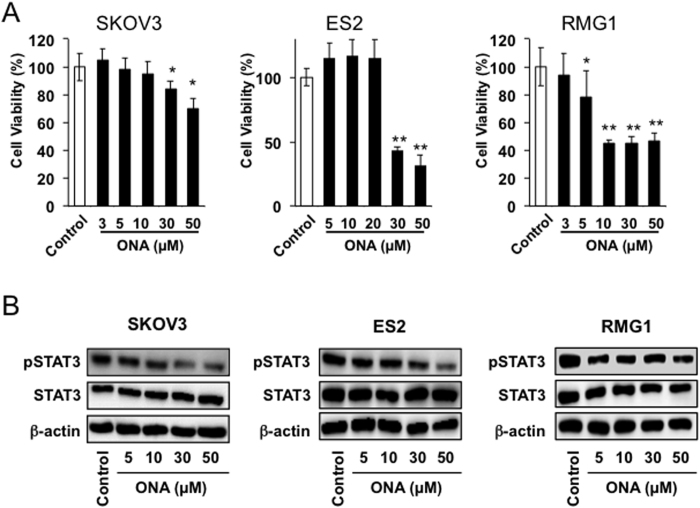
Effect of ONA on cell proliferation and STAT3 activation in EOC cells. EOC cells (SKOV3, ES2, and RMG1) were incubated with the indicated concentrations of ONA for 24 hours, followed by the determination of cell proliferation using the WST-8 assay (**A**). EOC cells were incubated with the indicated concentrations of ONA for 3 hours, followed by the determination of pSTAT3, STAT3 and β-actin by Western blot analysis, as described in the Materials and Methods (**B**). The data are presented as the mean ± SD. *p-value < 0.05, **p-value < 0.01 vs. control.

We next measured the effect of ONA on the proliferation of EOC cells (SKOV3, ES2 and RMG1) and found that ONA can significantly inhibit cancer cell proliferation at a concentration of at least 30 μM in SKOV3 (p-value < 0.05) and ES2 cells (p-value < 0.01) or 5 μM in RMG1 cells (p-value < 0.05) (Fig. 3A). As shown in Supporting Information 2, JAK2 and STAT3 activation was significantly suppressed by ONA treatment and slight inhibition of STAT1 and STAT6 activation was induced by ONA. Because STAT3 is known to be an important molecule involved in both M2 polarization and cancer cell proliferation, we hypothesized that ONA influenced STAT3 activation. As expected, STAT3 activation was significantly inhibited by ONA treatment in all cell lines (Fig. 3B). A similar phenomenon was observed when EOC cells were treated with a STAT3 inhibitor (WP1066) (Supporting Information 3), indicating that STAT3 activation is closely involved in EOC proliferation.

### ONA induces apoptosis and increases sensitivity against anti-cancer drugs in EOC cells

**Figure 4:**
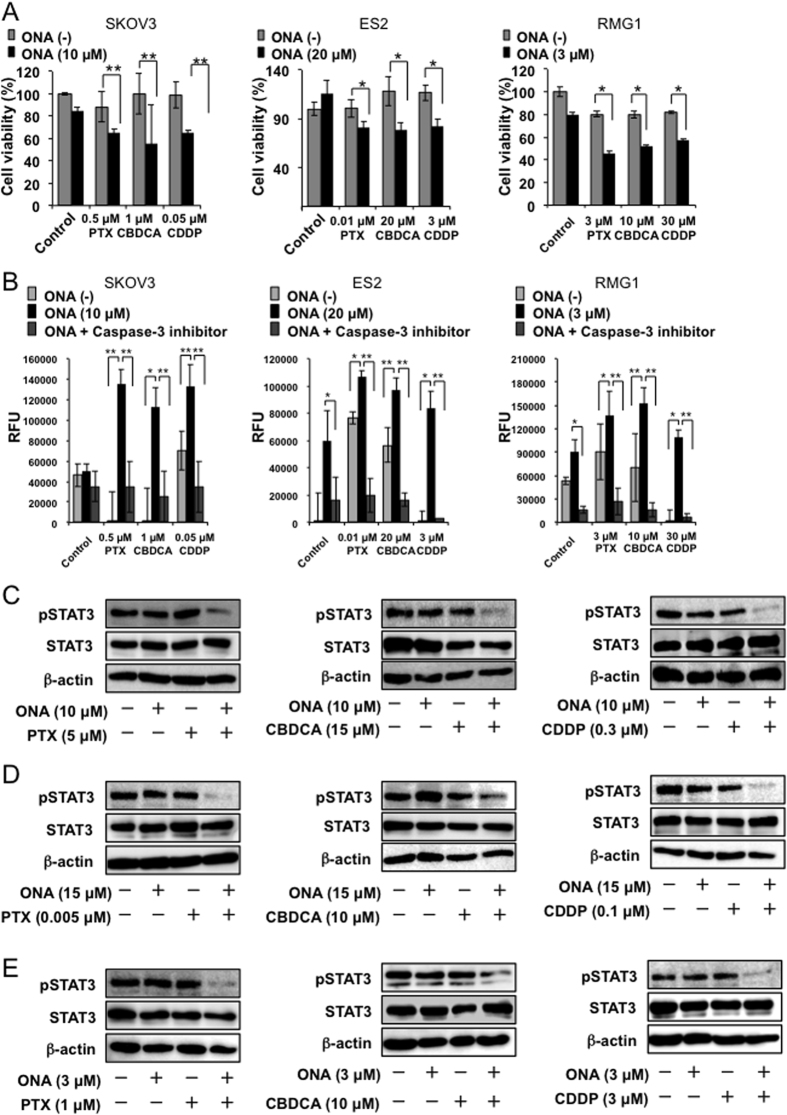
Combined effect of ONA and anti-cancer drugs in EOC cells. EOC cells (SKOV3, ES2, and RMG1) were incubated with a combination of both the ineffective concentration of individual anti-cancer drugs (PTX, CBDCA, or CDDP) and ONA for 24 hours, followed by the determination of cell proliferation using the WST-8 assay (**A**) and the ineffective concentration of each anti-cancer drug for each cell line was determined. In addition, the EOC cells were incubated with anti-cancer drugs and ONA for 4 hours, followed by caspase-3 measurement (**B**). The data are presented as the mean ± SD. *p-value < 0.05, **p-value < 0.01 vs. control (without anti-cancer drug). In addition, each EOC cell line (SKOV3: **C**, ES2: **D**, and RMG1: **E**) was incubated with an ineffective concentration of each anti-cancer drug (PTX, CBDCA and CDDP) with or without ONA for 3 hours, followed by the measurement of pSTAT3, STAT3 and β-actin by Western blot analysis, as described in the Materials and Methods.

To determine whether ONA enhances the anti-cancer effect of PTX, CBDCA and CDDP in EOC cells, the potential combinatorial effects with ONA were examined. First, we measured the maximum ineffective concentration of each anti-cancer drug on EOC cell proliferation. The maximum ineffective concentration of each anti-cancer drug is shown in Supporting Information 4. In the case of SKOV3 cells, 0.5 μM PTX, 1 μM CBDCA, and 0.05 μM CDDP did not have an inhibitory effect on cell proliferation (Supporting Information 4A). However, combined treatment with the ineffective concentrations of the anti-cancer drugs and ONA (10 μM) suppressed the proliferation of SKOV3 cells compared to treatment with single anti-cancer drugs (Fig. 4A). Similar results were observed in ES2 cells (Supporting Information 4B and Fig. 4A) and RMG1 cells (Supporting Information 4C and Fig. 4A). The combination index of ONA and the anti-cancer drugs was evaluated as previously reported^21^, and we found that ONA synergistically enhanced the anti-proliferative effect of the anti-cancer drugs (Supporting information 5).

In addition, we examined the effects of single and combined treatments on apoptosis to investigate the mechanism of the synergistic inhibitory effect induced by combined treatment with each anti-cancer drug and ONA. Caspase-3 activation in EOC cells was induced by single treatment with either ONA or the anti-cancer agents. Additionally, combined treatment with ONA and the anti-cancer agents upregulated caspase-3 activation compared to treatment with single anti-cancer agents (Fig. 4B). This upregulation was inhibited by a caspase-3 inhibitor, which supported the reliability of these experiments. Furthermore, STAT3 activation in EOC cells was also suppressed by combined treatment with ONA and each anti-cancer drug (Fig. 4C–E). These data demonstrated that ONA can enhance the cancer cell apoptosis induced by each anti-cancer drug via the suppression of STAT3 activation.

### ONA suppresses ovarian cancer development in murine models

**Figure 5:**
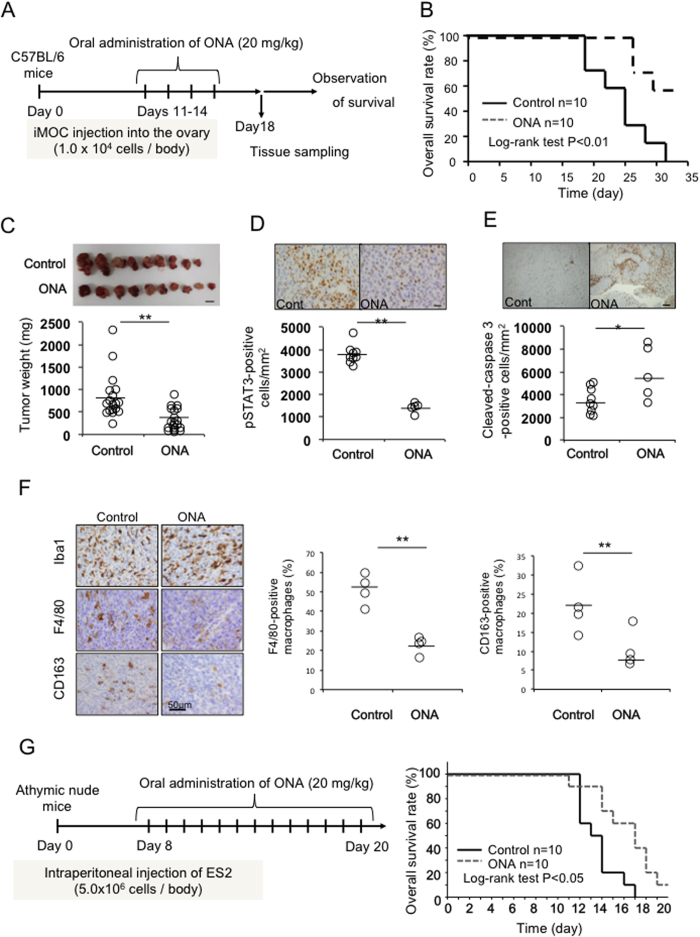
Effect of ONA on tumour progression in mouse models. As a murine ovarian cancer model, C57B6 mice were injected in the right ovary with iMOC cells and were administered ONA (20 mg/kg), as shown in the schematic diagram (**A**). Most of the untreated C57B6 mice died from cancer metastasis by day 40. The survival time (**B**) and tumour weight (**C**, scale bar: 1 cm) were evaluated. STAT3 activation (**D**, scale bar: 20 μm), caspase-3 activation (**E**, scale bar: 200 μm), and the infiltration of macrophages (**F**) in the tumour tissues were evaluated using immunostaining. The percentage of F4/80- and CD163-positive cells in Iba-1-positive macrophages was presented (**F**). Then, nude mice were injected in the intraperitoneal cavity with ES2 cells and were administered ONA (20 mg/kg), as shown in the schematic diagram (**G**) followed by the determination of the survival rate (**G**). Most of the untreated nude mice died from cancer metastasis by day 20.

We next evaluated the anti-cancer effect of ONA using an *in vivo* EOC model. As observed in human EOC cells, ONA inhibited the proliferation of iMOC cells by suppressing STAT3 activation (Supporting Information 6A,B). Furthermore, ONA also enhanced the inhibitory effect of CDDP on iMOC cell proliferation (Supporting Information 6C and D). We then investigated the effects of ONA in a murine ovarian cancer model. ONA was administered orally, as shown in Fig. 5A, and the results showed that ONA significantly prolonged survival (Fig. 5B) and suppressed tumour development (Fig. 5C). In addition, immunohistochemical studies were performed to investigate the effects of ONA, and STAT3 activation in the ovarian cancer tissue was decreased by the administration of ONA (Fig. 5D). Furthermore, cleaved-caspase 3-positive cells were increased in ONA-treated tumour tissues (Fig. 5E), thus suggesting that ONA can induce apoptosis in tumour cells. The infiltration of Iba-1^+^ pan-macrophages was not changed by ONA treatment; however, the percentages of F4/80^+^ and CD163^+^ macrophages were significantly reduced by ONA administration (Fig. 5F). On the other hand, the infiltration of CD4^+^ or CD8^+^ lymphocytes in cancer tissues was not altered by the administration of ONA (data not shown). These data indicated that ONA can suppress ovarian cancer development in murine models by inhibiting the M2 polarization of macrophages. Furthermore, cytotoxic effects in normal cells (HMDMs and normal lymphocytes) and side effects in animal models of ONA have not been observed (Supporting Information 7). ONA also inhibited human ovarian cancer (ES2) development and improved the survival rate in ES2-injected xenograft nude mice (Fig. 5G), thus suggesting that ONA can be an orally available small molecule for anti-ovarian cancer therapy.

### Combined effects of ONA and CDDP on ovarian cancer progression using an *in vivo* EOC model

**Figure 6:**
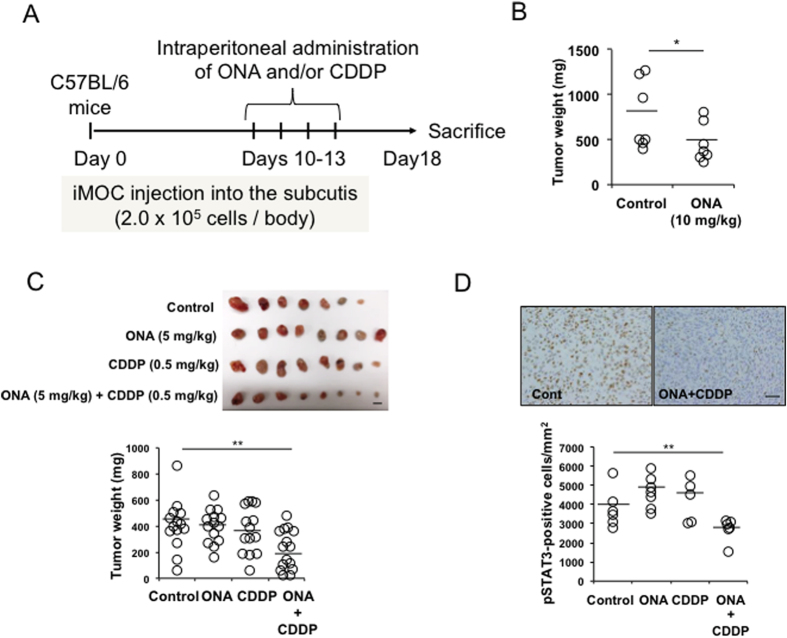
Combined effect of ONA and CDDP on tumour progression. As a subcutaneous tumour model, C57B6 mice were injected subcutaneously with iMOC cells and were administered the indicated concentrations of ONA and/or CDDP, as shown in the schematic diagram (**A**) followed by the determination of the tumour weight (**B,C**, scale bar: 1 cm). STAT3 activation in the subcutaneous tumour tissues was evaluated using immunostaining (**D**, scale bar: 50 μm). The data are presented as the mean ± SD. *p-value < 0.05, **p-value < 0.01 vs. control.

To clarify the potential of ONA as an auxiliary factor for anti-cancer therapy, we next investigated the combined effects of ONA and CDDP on tumour progression in the iMOC-bearing murine model, as shown in Fig. 6A. A single administration of ONA (10 mg/kg) significantly suppressed tumour development (Fig. 6B). On the other hand, a single administration of ONA (5 mg/kg) and CDDP (0.5 mg/kg) using a feeding tube had no effect on tumour progression, whereas the combined administration of these compounds (5 mg/kg ONA +0.5 mg/kg CDDP) significantly suppressed tumour progression compared to the control (Fig. 6C). Furthermore, the combined administration also decreased the number of pSTAT3-positive cells in the tumour tissues (Fig. 6D).

## Discussion

In this study, we demonstrated the anti-cancer effect of ONA and its combinatorial effect with CDDP *in vivo*. To the best of our knowledge, this study is the first to report an anti-ovarian cancer effect of ONA. We initially found that ONA inhibited the cell-cell interaction between M2 macrophages and cancer cells. We have previously demonstrated that cancer cell activation is induced by co-culture with M2 macrophages rather than M1 macrophages^6^. Here, we found that BrdU incorporation in cancer cells was increased by co-culture with control M2 macrophages rather than ONA-stimulated macrophages. The secretion of some cytokines by macrophages was found to be suppressed by ONA stimulation, indicating that the protumour activation of M2 macrophages was abrogated by ONA. Co-culture experiments were subsequently performed with low-dose ONA to investigate whether ONA could directly inhibit the cell-cell interaction between macrophages and cancer cells. The finding that ONA reduced BrdU incorporation in cancer cells co-cultured with M2 macrophages indicated that the cell-cell interaction was inhibited by ONA. Because STAT3 activation in co-cultured macrophages is significantly involved in cell-cell interaction^6^, the ONA-induced inhibition of the cell-cell interaction is considered to be due to STAT3 suppression by ONA. These observations suggested that the anti-cancer effect of ONA might be partially induced by the inhibition of direct contact-induced macrophage activation and STAT3 activation.

Many studies have revealed the detailed signalling mechanisms of macrophage differentiation, and we speculate that signalling molecules related to macrophage polarization towards the M2 phenotype are similar to those involved in cancer progression. STAT3 and NF-κβ are well known molecules involved in both macrophage differentiation into the M2 phenotype and cancer progression^22^. Because ONA inhibited macrophage activation and differentiation into the M2 phenotype, we hypothesized that ONA directly suppresses cancer cell proliferation. Consequently, cancer cell growth was significantly reduced and STAT3-activation was inhibited by ONA. These results suggested that ONA has a direct anti-cancer effect that suppresses cell proliferation in EOC cells, possibly by inhibiting STAT3 activation. In our preliminary data, NF-κβ activation was not changed by ONA (data not shown). Because STAT3 is involved in the proliferation and poor overall survival of EOC^23^, and blocking STAT3 signalling has been shown to suppress ovarian cancer progression in an experimental animal model^24^, STAT3 is considered a target molecule for treating patients with advanced EOC. STAT3 signalling has also been shown to be related to chemoresistance via the activation of stem cell properties in specific types of cancer cells, including EOC cells^25^,^26^. In the present study, we found that ONA treatment significantly led to the sensitization of EOC cells to the anti-cancer agents PTX, CBDCA and CDDP, which suggested that ONA potentially reduced the stem cell properties of EOC cells by inhibiting STAT3 signalling. ONA and anti-cancer agents were found to synergistically influence EOC cells (Supporting Information 5). STAT3 activation was significantly suppressed with combined treatment, as shown in Fig. 4, and this might indicate that the anti-cancer agents PTX, CBDCA and CDDP increased the sensitivity of EOC cells to ONA. On the other hand, ONA also slightly inhibited STAT1 and STAT5 activation (Supporting Information 2), thus suggesting that the other pathways besides STAT3 also might contribute to the anti-cancer therapeutic effects of ONA. These data indicated that the anti-cancer effect of anti-cancer drugs was enhanced by their combined administration with ONA and that the dose of these agents could be reduced by combined therapy with ONA, thus suggesting that ONA is a useful candidate as an auxiliary agent for anti-EOC therapy.

In conclusion, we demonstrated that ONA suppressed ovarian cancer progression by inhibiting the protumour functions of TAMs and reducing cancer cell proliferation. One of the anti-cancer mechanisms of ONA was revealed to be mediated by the inhibition of STAT3 activation, and ONA also enhanced the anti-cancer effect of anti-cancer drugs (Supporting Information 8). ONA is an orally available small molecule that may be an effective adjuvant therapy for patients with advanced EOC.

## Materials and Methods

### Cells and cell culture conditions

Three human ovarian cancer cell lines, SKOV3, ES2 and RMG1, were purchased from the American Type Culture Collection (Manassas, VA, USA), and a mouse ovarian cancer cell line (iMOC) was a kind gift from Professor Hideyuki Saya (Keio University, Tokyo, Japan)^27^. The cells were maintained in RPMI 1640 medium supplemented with 10% foetal bovine serum (FBS) (Gibco, Grand Island, NY) and regularly tested and found to be negative for *Mycoplasma* contamination.

Peripheral blood mononuclear cells were acquired from healthy volunteer donors; written informed consent for sample collection and subsequent analysis was obtained from all healthy donors. All protocols using human macrophages were approved by the Kumamoto University Hospital Review Board (No. 486), and conducted in accordance with the approved guidelines. CD14^+^ monocytes were purified from the peripheral blood mononuclear cells via positive selection using magnetic-activated cell sorting (Miltenyi Biotec, Bergisch Gladbach, Germany) and cultured with GM-CSF (10 ng/ml, Wako, Tokyo, Japan) or M-CSF (50 ng/ml, WAKO) for seven days to differentiate the cells into macrophages. The differentiated macrophages were then used as human monocyte-derived macrophages (HMDMs) in the present study, as described previously^28^.

### Flow cytometry

Macrophages were cultured on UpCell culture dishes (Cellseed, Tokyo, Japan) and stimulated with LPS (100 ng/mL, Sigma-Aldrich, St. Louis, MO, USA) or IL-10 (20 μg/mL, WAKO). Detached cells were then stained with mouse monoclonal anti-human CD163 (clone RM3/1) and anti-human PD-L1 (clone 10F.9G2) antibodies. Fc receptor Blocking Solution and all antibodies, including mouse monoclonal isotype-matched control antibodies, were obtained from BioLegend, Inc. (San Diego, CA, USA). The stained cell samples were analysed using a FACSverse™ machine and the FACSuite software programme (BD Biosciences, San Jose, CA, USA).

### Preparation of tumour culture supernatant (TCS)

ES2 cells were maintained in culture medium for 24 hours. The supernatant was used as a tumour culture supernatant (TCS) for this study.

### Extraction and isolation of ONA

Fresh, peeled onion bulbs were roughly chopped and blended in a mixer along with acetone, and the mixture was subsequently soaked in acetone for three days at room temperature. The filtrate was concentrated at 40 °C *in vacuo* to obtain a syrup residue, which was then subjected to passage over a polystyrene gel (Diaion HP-20), followed by elution with H_2_O and methanol. The eluted fraction with MeOH was subjected to silica gel column chromatography to yield ONA, as previously described^20^. Purified ONA was dissolved in dimethyl sulfoxide (DMSO) to create a 100 mM stock solution.

### Anti-cancer drugs

Paclitaxel (PTX; Nihon Kayaku, Tokyo, Japan), carboplatin (CBDCA; Wako), and cisplatin (CDDP; Wako) were dissolved in DMSO at the appropriate concentrations.

### Cytokine ELISA and cytokine array kit

HMDMs (3 × 10^5^ cells per well of a 12-well plate) were stimulated with LPS (100 ng/mL) for 24 hours after incubation with tumour cell supernatant (TCS) from SKOV3 cells or with TCS and ONA (30 mM) for 24 hours, followed by the determination of IL-10, IL-12 and TNF-α secretion by means of an ELISA kit (eBioscience, San Diego, CA, USA). The cytokine array kit was purchased from R&D Systems (Human XL Cytokine Array Kit #ARY022, Minneapolis, MN, USA).

### Cell proliferation and cytotoxicity assays

EOC cells (1 × 10^4^ cells) were cultured in a 96-well plate in quadruplicate before treatment. The cells were then cultured in the presence of ONA and/or the anti-cancer drugs. Cell viability was determined using a WST assay (WST-8 cell counting kit; Doujin Chemical, Kumamoto, Japan) according to the manufacturer’s protocol, and tumour cell proliferation was detected under co-culture conditions using a BrdU ELISA Kit (Roche, Penzberg, Bavaria, Germany).

EOC cells (1 × 10^5^ cells) were labelled with CytoTell Red (AAT Bioquest, Sunnyvale, CA, USA) and co-cultured with macrophages (1 × 10^5^ cells) for 2 days with or without ONA, and then cell proliferation was evaluated by flow cytometry.

### STAT3 activation assay

The degree of STAT3 activation was determined by measuring the increased expression of phosphorylated STAT3 (pSTAT3) with Western blot analysis, as previously described^15^. Briefly, the solubilized cells were run on a 10% SDS-polyacrylamide gel and transferred to a polyvinylidene fluoride (PVDF) transfer membrane (Millipore, Bedford, MA, USA). To detect pSTAT3, the membranes were exposed to an anti-pSTAT3 antibody (D3A7; Cell Signaling Technology Japan, Tokyo, Japan) and visualized using a horseradish peroxidase-conjugated anti-rabbit IgG antibody with ECL Western blotting detection reagent (GE Healthcare Life Sciences, Piscataway, NJ, USA). To detect STAT3, the membranes were exposed to anti-STAT3 antibody (sc-8019; Santa Cruz Biotechnology, Inc., Santa Cruz, CA, USA) and visualized using a horseradish peroxidase-conjugated anti-mouse IgG antibody with ECL Western blotting detection reagent. The membranes were re-blotted with an anti-β-actin antibody (C4) (sc-47778; Santa Cruz Biotechnology, Inc.) as an internal calibration control.

### Fluorimetric cleaved caspase-3 assay

Caspase-3 activities were measured using the Amplite^TM^ Fluorimetric Caspase 3/7 Assay Kit (AAT Bioquest, Inc., Sunnyvale, CA, USA) according to the manufacturer’s instructions. Briefly, 1 × 10^4^ EOC cells were cultured in 96-well plates and ONA and/or anti-cancer drugs were added to the cells. After 24 hours, the caspase-3 activity was determined.

### Murine ovarian cancer model and subcutaneous model

Female C57BL/6 mice (6–8 weeks) were purchased from CLEA Japan (Tokyo, Japan). We used iMOC cells for the *in vitro* and *in vivo* studies. iMOC cells were suspended in 100 μL of RPMI and injected in the ovary (1 × 10^4^ cells) or subcutaneously injected (2 × 10^5^ cells). In the ovarian cancer model, ONA or vehicle (0.5% methylcellulose) was administered orally using a feeding tube, and ONA, CDDP or vehicle (0.5% methylcellulose) was administered intraperitoneally in the subcutaneously implanted model on days 10–13 after iMOC injection. The mice were sacrificed on day 18, followed by the determination of the weight of ovarian tumours or subcutaneous cancer development.

Female athymic nude mice (6–8 weeks) were purchased from CLEA Japan. ES2 cells (5 × 10^6^ cells/mouse) were suspended in 100 μL of RPMI and injected into the intraperitoneal cavity. ONA or vehicle (DMSO) was administered orally on days 8–20 after ES2 injection, followed by the determination of the survival rates. All animal experiments were approved by the Ethics Committee for Animal Experiments of Kumamoto University (Permission Number: B24-125) and conducted in accordance with the Guidelines for Animal Experiments of Kumamoto University.

### Immunohistochemistry

Murine ovarian cancer or subcutaneous cancer tissue specimens were fixed in paraformaldehyde and then embedded in paraffin. After sectioning (3 μm thick), paraffin-embedded cancer tissues were used for immunostaining with anti-pSTAT3 (D3A7; Cell Signaling Technology), anti-Iba-1 (WAKO), anti-F4/80 (clone CI:A3-1, Serotec, Ltd., Oxford, UK), anti-CD163 (rabbit polyclonal, Cosmo Bio, Tokyo, Japan), anti-Ki67 (DAKO, Glostrup, Denmark), anti-cleaved caspase-3 (5A1E; Cell Signaling Technology, Beverly, MA, USA) and anti-CD3 (Abcam, Cambridge, UK) antibodies.

Murine tissues were also embedded in Tissue-Tek OCT (Sakura Finetek, Torrance, CA, USA), snap-frozen in liquid nitrogen and stored at −80 °C. After sectioning (7 μm thick), the tissues were fixed with cold acetone and treated with the following primary antibodies: anti-CD4 (GK1.5; ATCC, Manassas, VA, USA) and anti-CD8 (53–6.72; ATCC, Manassas, VA, USA). The sections were subsequently treated with an HRP-conjugated secondary antibody (Nichirei, Tokyo, Japan), and the reactions were visualized with diaminobenzidine. The number of immunopositive cells was counted using the BZ-9000 analysis software programme (Keyence, Osaka, Japan).

### Statistics

All data are representative of two or three independent experiments. The data are expressed as the mean ± standard deviation (SD). Differences between the groups were examined for statistical significance using the Mann-Whitney U-test and a non-repeated measures ANOVA. A Kaplan-Meier analysis was performed to assess the murine overall survival and compared using the log-rank test. A p-value of <0.05 was considered to indicate the presence of a statistically significant difference.

## Additional Information

**How to cite this article**: Tsuboki, J. *et al*. Onionin A inhibits ovarian cancer progression by suppressing cancer cell proliferation and the protumour function of macrophages. *Sci. Rep.* **6**, 29588; doi: 10.1038/srep29588 (2016).

## Supplementary Material

Supplementary Information
