# Supplementary material for: Onionin A inhibits ovarian cancer progression by suppressing cancer cell proliferation and the protumour function of macrophages
Source: Sci Rep. 2016 Jul 12;6:29588. doi: 10.1038/srep29588 (PMC4941721; doi:10.1038/srep29588)
Supplement: Supplementary Information [file srep29588-s1.pdf]

# Onionin A inhibits ovarian cancer progression by suppressing cancer cell proliferation and the protumour function of macrophages.

Junko Tsuboki[1, 2]*, Yukio Fujiwara[1]*, Hasita Horlad[1], Daisuke Shiraishi[1], Toshihiro Nohara[3], Shingo Tayama[2], Takeshi Motohara[2], Yoichi Saito[1], Tsuyoshi Ikeda[3], Kiyomi Takaishi[2], Hironori Tashiro[4], Yukihiro Yonemoto[5], Hidetaka Katabuchi[2], Motohiro Takeya[1], Yoshihiro Komohara[1]

[1]Departments of Cell Pathology, and [2]Obsterics and Gynecology, Graduate School of Medical Sciences, Kumamoto University, Honjo 1-1-1, Chuo-ku, Kumamoto 860-8556, Kumamoto, Japan. [3]Department of Natural Medicine, Faculty of Pharmaceutical Sciences, Sojo University, Ikeda 4-22-1, Nishi-ku, Kumamoto 860-0082, Japan. [4]Department of Mother-Child Nursing, Faculty of Life Sciences, Kumamoto University, Kuhonji 4-24-1, Chuo-ku, Kumamoto 862-0976, Kumamoto, Japan. [5]Priority Organization for Innovation and Excellence, Kumamoto University, Kurokami 2-39-1, Chuo-ku, Kumamoto 860-8555, Kumamoto, Japan

* These authors contributed equally to this work

**Address for correspondence:** Yoshihiro Komohara, M.D., Ph.D.,Department of Cell Pathology, Graduate School of Medical Sciences, Kumamoto University, Honjo 1-1-1, Chuo-ku, Kumamoto 860-8556, Kumamoto, Japan. Phone: +81-96-373-5095. Fax: +81-96-373-5096. E-mail: ycomo@kumamoto-u.ac.jp

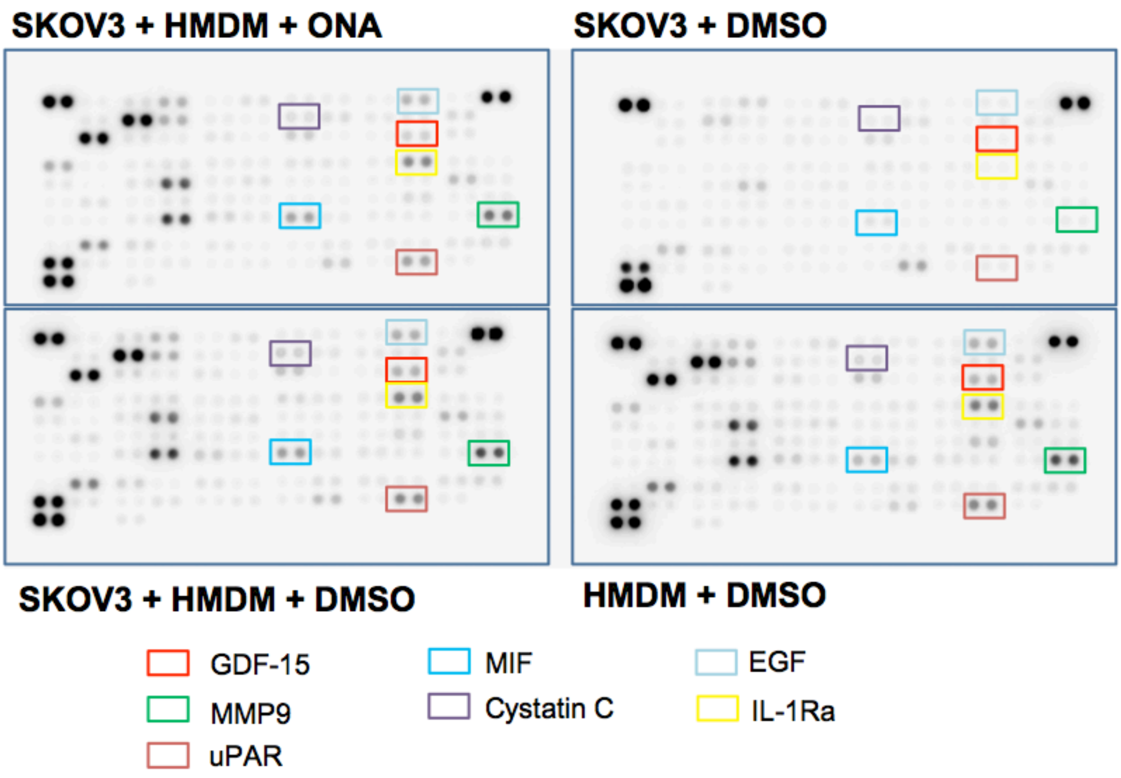


**Supplementary Information 1.** ***Cytokine array of culture supernatant.*** SKOV3 cells were incubated with HMDM during treatment of ONA (10 μM) for 24 hours, followed by the determination of cytokines production in culture supernatant by cytokine array kit.

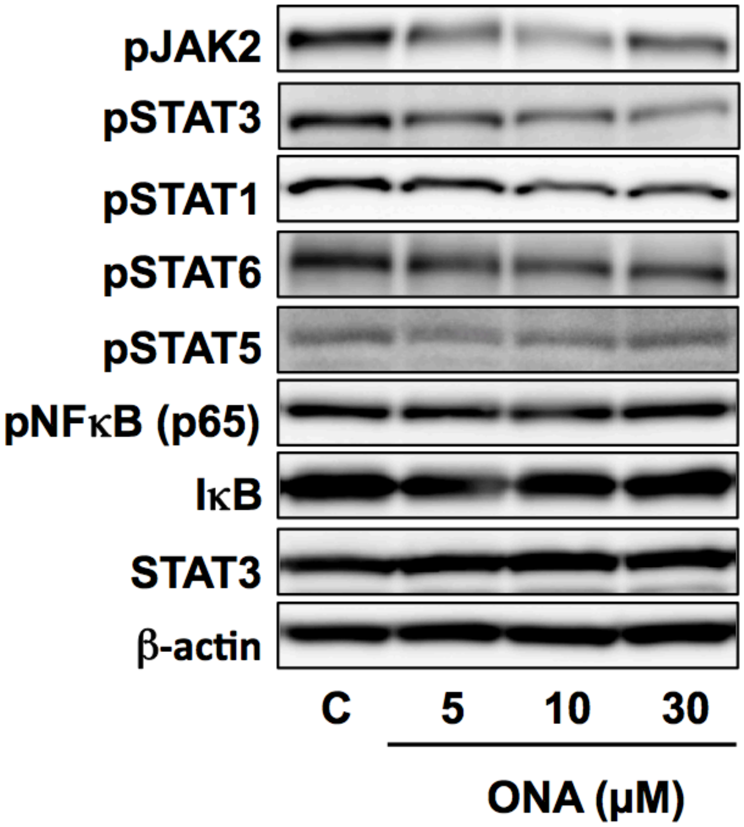


**Supplementary Information 2.** ***Effect of ONA on JAK, STAT and NF-kB activation in SKOV3 cells.*** SKOV3 cells were incubated with the indicated concentrations of ONA for 4 hours, followed by the determination of pJAK2, pSTAT1, pSTAT3, pSTAT5, STAT6, pNF-κB, IκB, STAT3 and β-actin by a Western blot analysis.

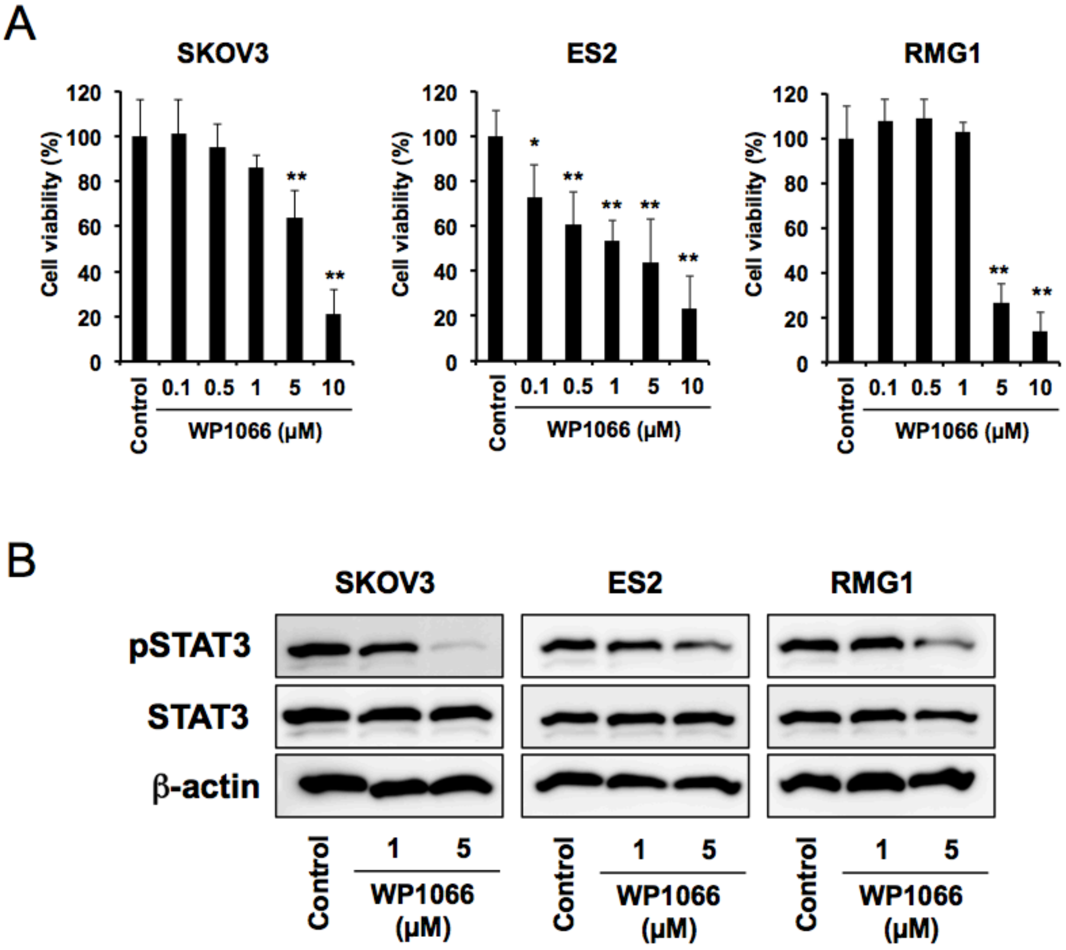


**Supplementary Information 3.** ***Effect of WP1066 on cell proliferation and STAT3 activation in EOC cells.*** EOC cells (SKOV3, ES2, and RMG1) were incubated with the indicated concentrations of WP1066 for 24 hours, followed by the determination of cell proliferation using the WST-8 assay (A). EOC cells were incubated with the indicated concentrations of ONA for 3 hours, followed by the determination of pSTAT3, STAT3 and β-actin by a Western blot analysis (B). The data are presented as the mean±SD. *: p-value <0.05, **: p-value <0.01 vs. control.

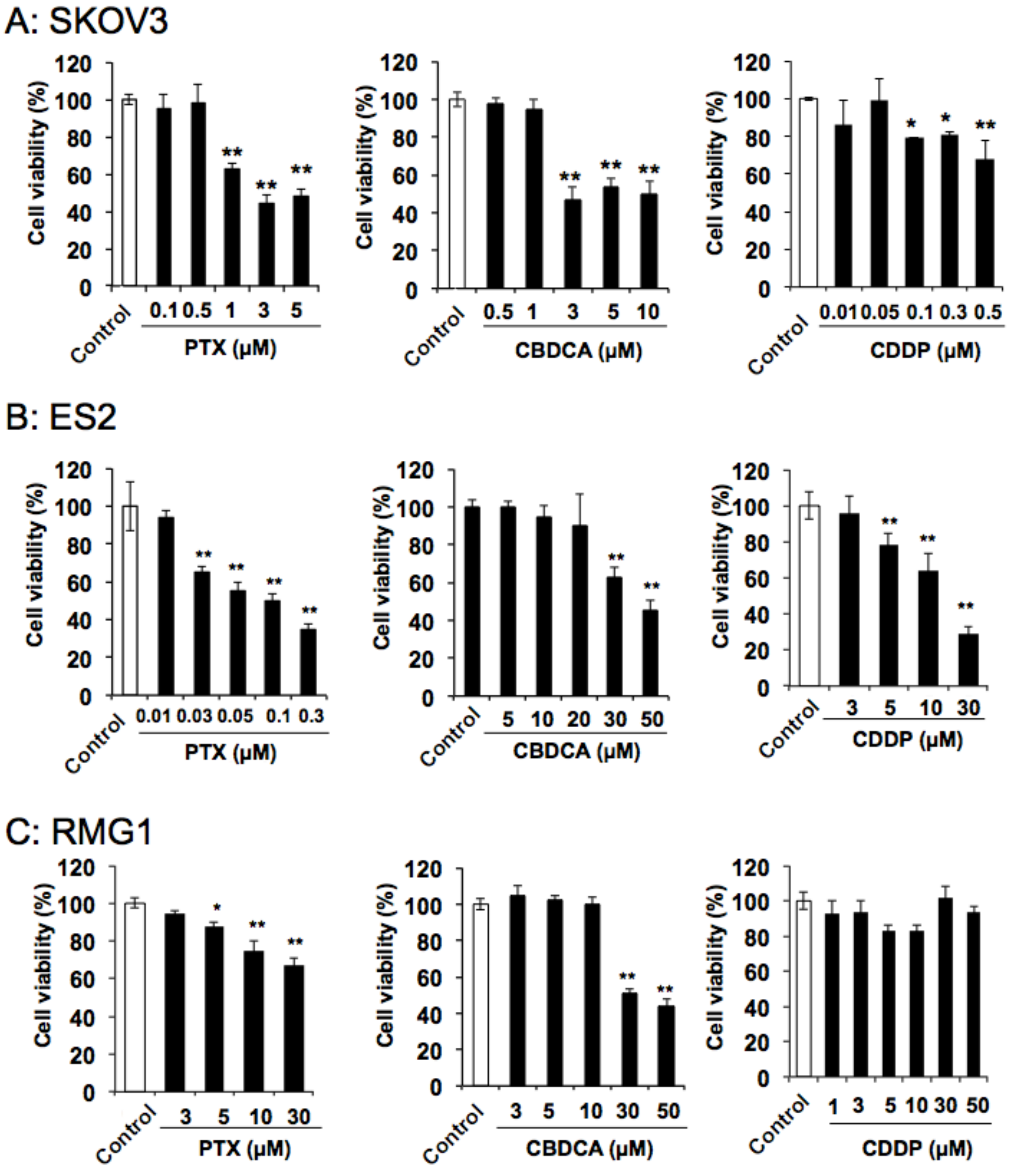


**Supplementary Information 4.** ***Ineffective concentration of ONA and anti-cancer drugs on cell proliferation.*** EOC cells (SKOV3, ES2, and RMG1) were incubated with the indicated concentrations of PTX, CBDCA, and CDDP for 24 hours, followed by the determination of cell proliferation using the WST-8 assay (SKOV3: A, ES2: B, RMG1: C), and the ineffective concentration of each anti-cancer drug on each cell line was determined. The data are presented as the mean±SD. *: p-value <0.05, **: p-value <0.01 vs. control.

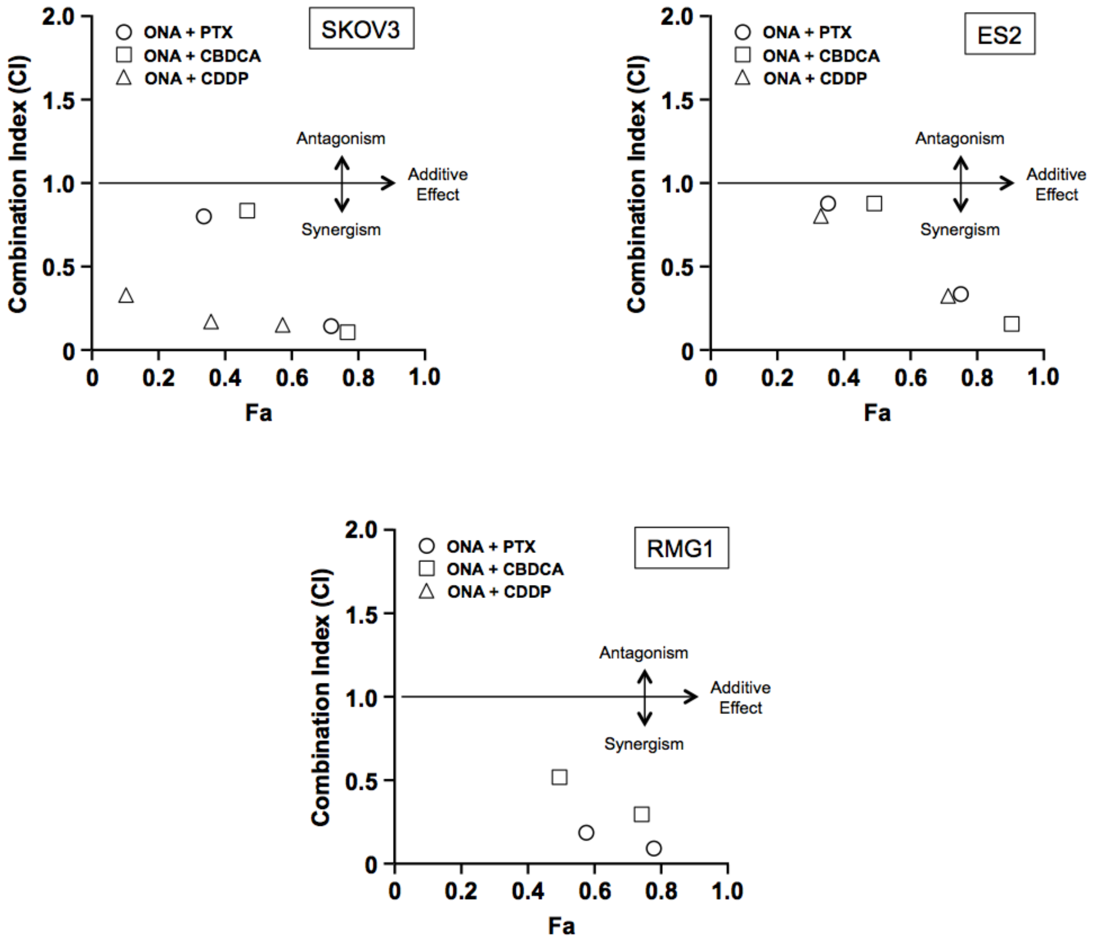


**Supplementary Information 5.** ***The fraction affected (Fa)-combination index (CI) plot (Chou-Talalay Plot).*** The effect relationships of ONA and anti-cancer drugs (PTX, CBDCA, or CDDP) and two-drug combinations on growth inhibition in EOC cells (SKOV3, ES2 and RMG1). The *fa*-CI plot with x=fraction affected (Fa) vs. y=combination index (CI) where CI <1, =1, and >1 indicates synergism, additive effect and antagonism, respectively.

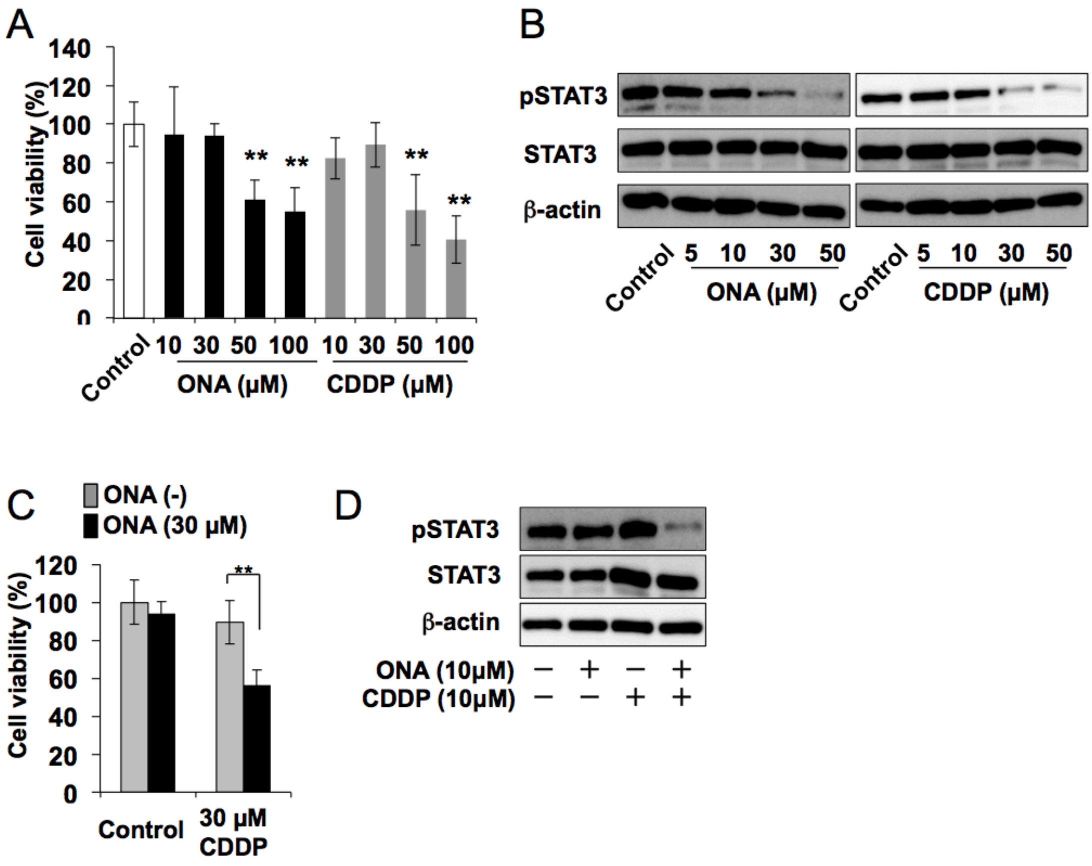


**Supplementary Information 6.** ***Effect of ONA on murine ovarian cancer cell line.*** iMOC cells were incubated with the indicated concentrations of ONA or CDDP for 24 hours, followed by the determination of cell proliferation using the WST-8 assay (A). iMOC cells were incubated with the indicated concentrations of ONA and/or CDDP for 3 hours, followed by the determination of pSTAT3, STAT3 and β-actin by a Western blot analysis (B). iMOC cells were incubated with a combination of the ineffective concentration of anti-cancer drug and ONA, followed by the determination of cell proliferation using the WST-8 assay (C). iMOC cells were incubated with the ineffective concentration of each anti-cancer drug with or without ONA for 3 hours, followed by the determination of pSTAT3, STAT3 and β-actin by a Western blot analysis (D).

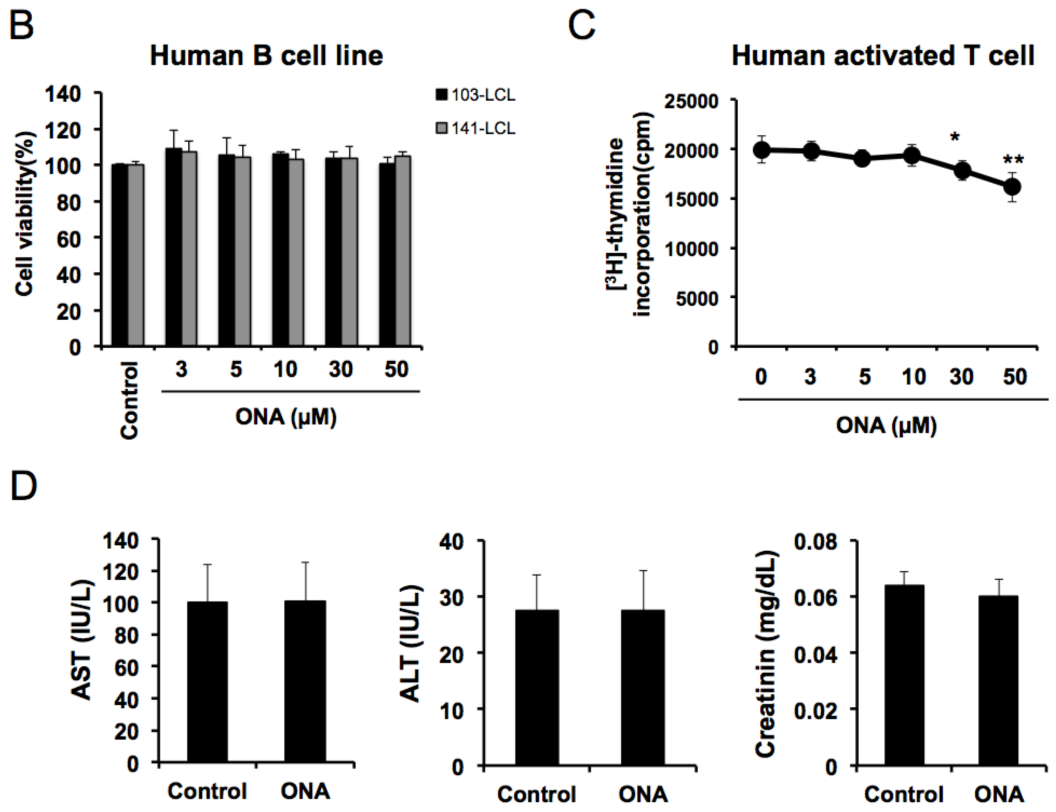


**Supplementary Information 7.** ***Effect of ONA on cytotoxicity in normal cells and mice.*** Human monocyte-derived macrophages (HMDMs) were treated with ONA for 24 hours and cell viability was tested by WST assay (A). EBV transformed B-lymphoblast cell line (103-LCL, 104-LCL) were treated with ONA for 24 hours and cell viability was tested by WST assay (B). PBMCs from two donors were mixed at 1:1 ratio following cultured for 5days with or without ONA, and the 3H-thymidine incorporation assay was performed (C). ONA (60 mg/kg) was administrated to mice fourth a week for 2 weeks, followed by determination of AST, ALT and creatinin concentration in blood (D). The data are presented as the mean±SD. *: p-value <0.05, **: p-value <0.01 vs. control.

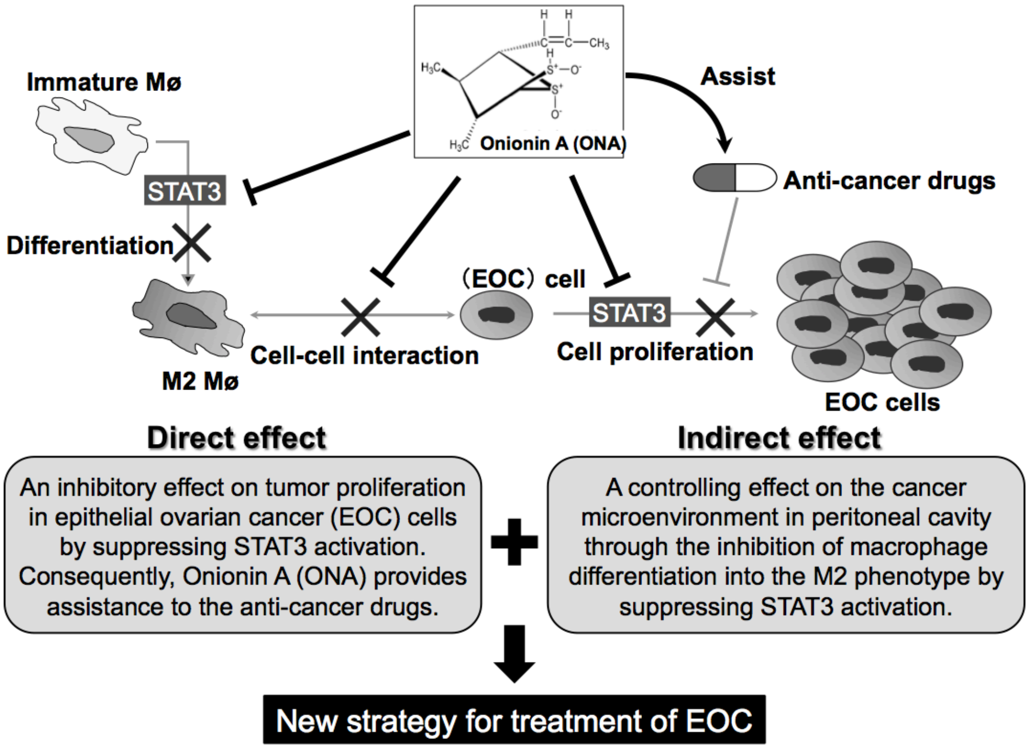


**Supplementary Information 8.** ***Inhibitory mechanism of ONA on EOC progression.*** As shown in the schematic diagram, ONA has indirect and direct anti-cancer effects. Indirect effect is to control differentiation into M2-polarization of macrophages that support EOC progression. Direct effect includes to inhibit EOC cells' proliferation and to assist anti-cancer effect of anti-cancer drugs.
